# Supplementary material for: Detection of Fused Genes in Eukaryotic Genomes using Gene deFuser: Analysis of the Tetrahymena thermophila genome
Source: BMC Bioinformatics. 2011 Jul 11;12:279. doi: 10.1186/1471-2105-12-279 (PMC3143110; doi:10.1186/1471-2105-12-279)
Supplement: Additional file 1 — Results of Gene deFuser for the Tetrahymena thermophila genome. This zip file contains the raw results of the analysis of the Tetrahymena genome using Gene deFuser. To view the contents, unzip the file and open the Final_Tet.html file in the resulting folder. [file 1471-2105-12-279-S1.ZIP › Results/22.m00350.html]

Gene deFuser -- Results of Job Final\_Tet

 


Gene deFuser

| Home | Retrieve Results | References | Help |
| --- | --- | --- | --- |

Back to Main Results of Job Final\_Tet

# Query Name: 22.m00350

Candidate fusion gene

## Query Sequence:

MENSNVSKKLERIIQLLKDKQHLTVVNPNAGSGAYGQVMTAKEENGEEVAIKIVSVFDEIDSIDEQKLKEIKSEAQFLQMCHHPNIVSYKGEFQLGSCYFIKMEKGEMSLQEFVDKKLNQGMSEKLFVKLALQMLSAIQYLHSKNYVLRDISLKNVLLDKDYNIKLCDFGLAKEINPQLATQAFLQSQVKGVLFYFPPEVLVMTNFNSEEKKSQKIYQNFDGDVWAFGVCMYLLGGASFLHVLQLTQGKETFQLSKLVSIEINQILLNTLQLDPKLRPSIDKIIQDFKNIQLNLEKGKDDEIGYYFNQTELFQQAQKEIDNHKYQEAQVTLERLVKINTHNDQYLALLGLTHLEQYQCEESRSISQKCLSINPKNEIALSCMGYYYYEKNDLKQAMCYLQKCLNLNPKNYRALTYKAFVLSNQQKLDEELLTLKEAISYNQNYPYSILQIGKCYFKKKMYEDAITSFKQVIKLLPTIFSPYYCLGIIYYERSEFDQSISYFNKALELNSSNQNCLYSLAKVYLETFNYSNAIEILNTLVRLFPSNDLYLATLALAYSEIKDEQKVMEFSQKSLQINPQNILALNCLAYYYFLKQQNQLAIQCLQSSIKVNPCNFRAYFYQAQILLSEGKHEDAILSAKYSMQQNSKFPNAQFLLGQVFEQIGKMDKAIKYFQEAIMIQAKYELPYIDLSQIYRKKGQYDDALFICKLALQNNIKSAKIYNELGKIYEKKQYLSQAINYFSECISLKPSEQIYQNNLKNCKELLSKSNSKIKI

### Significant Ortholog Group Hits and their Scores:

| N terminus | | C terminus | |
| --- | --- | --- | --- |
| [R] KOG0611 Predicted serine/threonine protein kinase | 32.9996385066613 | [GOT] KOG4626 O-linked N-acetylglucosamine transferase OGT | 32.4983777386932 |
| [T] KOG0576 Mitogen-activated protein kinase kinase kinase kinase (MAP4K), germinal center kinase family | 29.7881906112285 |  |  |
| [T] KOG0574 STE20-like serine/threonine kinase MST | 29.5330962165735 |  |  |
| [T] KOG4236 Serine/threonine protein kinase PKC mu/PKD and related proteins | 27.1742844729082 |  |  |
| [T] KOG0690 Serine/threonine protein kinase | 20.5044406905194 |  |  |
| [R] KOG0597 Serine-threonine protein kinase FUSED | 18.8953174281028 |  |  |
| [T] KOG4721 Serine/threonine protein kinase, contains leucine zipper domain | 18.6487697739932 |  |  |
| [T] KOG0201 Serine/threonine protein kinase | 18.5685186278108 |  |  |
| [T] KOG0579 Ste20-like serine/threonine protein kinase | 16.0006475610433 |  |  |
| [R] KOG0589 Serine/threonine protein kinase | 14.4770629639671 |  |  |
| [T] KOG0577 Serine/threonine protein kinase | 14.2737873121932 |  |  |
| [D] KOG0615 Serine/threonine protein kinase Chk2 and related proteins | 13.2517170814561 |  |  |
| [R] KOG0586 Serine/threonine protein kinase | 11.7717127202183 |  |  |
| [T] KOG0578 p21-activated serine/threonine protein kinase | 9.49470789399227 |  |  |
| [G] KOG0599 Phosphorylase kinase gamma subunit | 8.32265660354373 |  |  |
| [T] KOG0198 MEKK and related serine/threonine protein kinases | 8.02975437953431 |  |  |
| [T] KOG4717 Serine/threonine protein kinase | 7.13681961473225 |  |  |
| [T] KOG0587 Traf2- and Nck-interacting kinase and related germinal center kinase (GCK) family protein kinases | 6.86443043728833 |  |  |
| [UT] KOG0662 Cyclin-dependent kinase CDK5 | 6.73447401433335 |  |  |
| [T] KOG0583 Serine/threonine protein kinase | 6.40980971865717 |  |  |
| [R] KOG0594 Protein kinase PCTAIRE and related kinases | 6.13837325053822 |  |  |
| [D] KOG0575 Polo-like serine/threonine protein kinase | 5.60730134915464 |  |  |

#### Graphs (click to enlarge):

|  |  |
| --- | --- |
| BLAST of Query Sequence | Location of Ortholog Group Hits |
|  |  |

Contact: Andre Cavalcanti\_\_\_\_\_Last Modified September 14, 2010
